# Migration of Intraocular Silicone Oil to CNS: The Role of Elevated Intraocular Pressure and Congenital Optic Nerve Abnormalities

**DOI:** 10.1007/s00062-015-0460-5

**Published:** 2015-09-07

**Authors:** A. Grzybowski, F. J. Ascaso

**Affiliations:** 1 Department of Ophthalmology, Poznan City Hospital, 3 Szwajcarska St, 361-285 Poznań, Poland; 2 Chair of Ophthalmology, University of Warmia and Mazury, Olsztyn, Poland; 3 Medical and Surgical Retina Unit, Department of Ophthalmology, Hospital Clínico Universitario Lozano Blesa, Zaragoza, Spain; 4 School of Medicine, University of Zaragoza, Zaragoza, Spain; 5 Instituto de Investigación Sanitaria de Aragón (IIS Aragón), Zaragoza, Spain

Dear Editor,

We have read with interest the article “Intracranial Migration of Intravitreal Silicone Oil: A Case Report” published by Kim et al. [1]. We would like to congratulate them for the phantom study of silicon oil (SiO) and perfluorocarbon liquid (PFCL). We believe, however, that some discussion of this article is needed. In fact, we must clarify that, although both types of intraocular tamponade are used today, SiO is a long-term intraocular tamponade, whereas PFCL is only used as intraoperative tool and must be extracted at the end of surgery. We agree with the authors that the increased intraocular pressure of the patient may have caused intracranial migration of SiO [2]. However, they provide no information about either the evolution during the 9 months of follow-up or the presence of congenital optic nerve disc anomalies. Finally, we also believe that the article should include the characteristics of the SiO employed. These clinical data regarding the presence of any of these predisposing factors in the patient might have played a crucial role in physical silicone migration.

## Conflict of Interest

The authors have no conflict of interest.
